# Addressing Suicide in the Veteran Population: Engaging a Public Health Approach

**DOI:** 10.3389/fpsyt.2020.569069

**Published:** 2020-11-23

**Authors:** David Carroll, Lisa K. Kearney, Matthew A. Miller

**Affiliations:** United States Department of Veterans Affairs, Washington, DC, United States

**Keywords:** suicide prevention, suicide intervention, Public health approach, veterans, Department of Veterans Affairs

## Abstract

Suicide is a national public health issue in America, and it disproportionately affects those who are serving or who have served in the United States military. The US Department of Veterans Affairs (VA) has made suicide prevention its number one clinical priority. VA is committed to prevent suicide among the entire population of those who have served our country in the military, regardless of whether they make use of any VA services or benefits. Suicide can be prevented through the application of a public health strategy embracing partners at all levels. Following a national strategy, VA has embarked on an effort involving the application of a public health strategy combining both clinically-based and community-focused interventions. This paper describes several examples of these efforts and steps forward.

## Introduction

In 2017, 6,139 veterans died by suicide (1). These veterans were among the over 45,000 Americans who died by suicide during that same year. Suicide is not caused by any one factor, nor can suicide be prevented by application of any one strategy (2, 3). While mental health concerns comprise particular risk factors for suicide, larger societal issues also serve as additional unique risk factors for suicide (e.g., homelessness, financial concerns, relationship distress, unemployment, increasing alcohol sales, and increasing sales of and access to firearms). Our national strategies must not only include clinically-based intervention strategies, but also proactive community-based prevention efforts to also address these broader factors. In 2018, VA published a national strategy for preventing Veteran suicide (4) followed in 2019 by the publication of the revised VA-DoD Clinical Practice Guideline on the Assessment and Management of Patients at Risk for Suicide (5). These documents provide overarching guidance for the vision and implementation of VA's national suicide prevention initiative.

## What we Know From the Data

Since 2014, VA has published an annual report on suicide death data among veterans. The primary purpose of veteran suicide data reporting is to provide critical information to move suicide prevention efforts forward. The VA creates state data sheets as a companion to the national report to prompt local/ regional action (6). The annual report is based upon a close collaboration with the Department of Defense (DoD) and the Centers for Disease Control and Prevention (CDC). VA and DoD partners complete searches of the National Center for Health Statistics' National Death Index (NDI), the national gold standard of all individuals who have died to identify all individuals who are veterans, which is compiled annually by the CDC based-upon national suicide mortality data reported to the CDC by each state and US territory. NDI data is typically released 11 months after the end of a calendar year and then followed by this extensive detailed review for veterans, which takes significant time to ensure information accuracy. For example, the 2019 National Suicide Prevention Annual Report was published in September 2019, and it included the suicide death data for Veterans between 2005 and 2017 with the reporting period ending on December 31, 2017 (1).

The report contains information on counts, measures of central tendency, and rates broken down by age, gender, means of death, and a few additional key variables. In 2005, an average of 87 American adults (including an average of 16 veterans) died by suicide each day. In 2017, an average of 124 Americans died by suicide each day (including an average of 17 veterans). However, given the decline in the veteran population during that time period, the suicide rate for veterans in 2017 was 1.5 times higher than the rate for non-veteran adults (2.2 times higher among female veterans than non-veteran women, and 1.3 times higher among male veterans than non-veteran males). The highest rate of suicide among veterans is among male veterans between the ages of 18 and 34, but the highest number of suicides among veterans is among male veterans age 55 and older. Nearly 70% of veteran suicide deaths (69.4%) resulted from a firearm injury which is higher than among non-veteran adult suicide deaths (48.1%).

## Foundational Components of Va's Suicide Prevention Initiative: The National Strategy, tHE VA/DoD Clinical Practice Guideline, and Research

National Strategy for Preventing Veteran Suicide (4) provides a framework for identifying priorities, organizing efforts, and leading a national effort to prevent veteran suicide over the next decade. It aligns with the 2012 National Strategy for Suicide Prevention and the strategy published by the Department of Defense. The National Strategy contains four strategic directions, 14 goals, and 43 specific objectives, all framed within a public health approach. The national strategy is built upon the National Academy of Medicine model of having actions focused on the entire population (Universal), those known to be at higher risk (Selective), and those known to be at highest risk (Indicated). The national strategy leverages the systems within which veterans typically live—families, communities, healthcare systems, workplaces, schools, faith-based and other social groups—to ensure all veterans are reached, both inside and outside of the VA system.

In 2013, the VA and DoD published the first clinical practice guideline (CPG) for the assessment and management of patients at risk for suicide. A revised and updated CPG, the VA/DoD Clinical Practice Guideline for the Assessment and Management of Patients at Risk for Suicide, was published in 2019 and is based upon a thorough review of the existing literature at the time of publication (5). The CPG is intended to guide clinical decision-making at critical points in the identification and management of suicidal behavior. The CPG identifies essential features and potential actions for both those at acute risk and at chronic risk. The CPG contains five recommendations on screening and evaluation, including three that VA currently uses as part of its comprehensive suicide risk screening and evaluation program. It also contains 12 recommendations on risk management and treatment, including several items that VA currently implements at its health care facilities.

## Va's Public Health Approach

**Figure 1:**
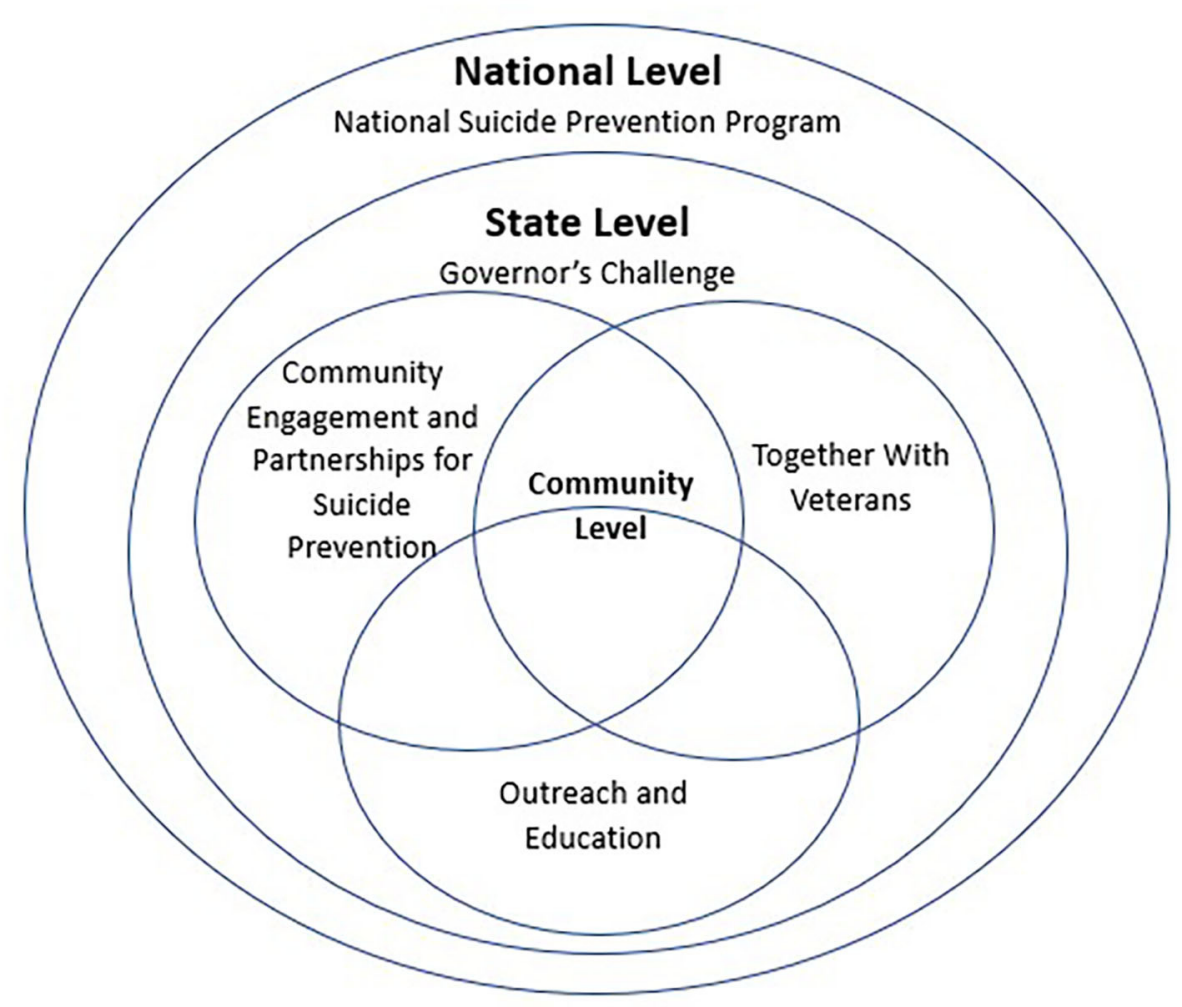
National, state, community program coordination.

To accomplish VA's goal of reducing suicide among all 20 million U.S. Veterans, a comprehensive public health approach that blends clinically-based interventions and community-based prevention strategies is needed (See Figure 1, National, State, Community Program Coordination). VA is currently deploying both strategies, with high level examples described below, to ensure the fullest implementation of the public health approach to suicide prevention across the nation.

## Examples of Va's Clinically-Based Interventions

Promoting evidence-based clinical strategies are a key component to suicide prevention. Clinically-based strategies rely upon a foundational level of staffing to ensure success. Mental health staff enhancements have been associated with decreases in suicide rates among VHA patients in regions where the increases in mental health outpatient staffing were greatest (7). In order to promote maximum success of clinically-based interventions for suicide prevention in alignment with the evidence-base, VA is striving to reach recommended staffing levels. This includes a minimum outpatient mental health staffing ratio of 7.72 outpatient mental health full time employee equivalent (FTE) staff to 1,000 veterans in outpatient mental health and a national minimum benchmark for suicide prevention staffing at 0.1 suicide prevention coordinators/case manager FTEE per 1,000 veterans enrolled at a facility. Below we outline a few examples of clinically-based interventions deployed nationally in VA as part of the strategic plan for suicide prevention.

### Universal Screening

Identifying veterans at risk for suicide prior to a time of crisis is a critical factor in the deployment of VA's national strategy. The implementation of universal screening for suicide has a strong evidence-base (5). In 2018, VA launched the largest standardized suicide risk screening and assessment process in the country, known as the Suicide Risk Identification Strategy (Suicide Risk ID) which includes a first and second level screens followed by a comprehensive suicide risk evaluation as indicated. The population-based mental health screening process is implemented for those with unrecognized risk (universal), for those who may be at risk (selected), and for those at elevated risk (indicated). When initial screening is positive, veterans are provided with a comprehensive suicide risk evaluation. For veterans presenting to other VHA services VA has setting specific guidance for screening and assessment. Between October 1, 2018, and March 2, 2020, 4,533,105 veterans have been screened for suicide across the VHA in all ambulatory settings. Veterans identified at risk through this process are then connected with services to get them the care they need when they need it.

### Safety Planning in the Emergency Department

One advantage of working in a large health care system is the ability to deploy innovation at a rapid pace. One such example is the deployment of Safety Planning in Emergency Departments (SPED). National VA leaders were inspired about initial research findings on safety planning and follow-up caring contacts for those seen in emergency department settings (8). Through implementation of this program, Stanley et al. (8) found a reduction in suicidal behaviors by almost half (45%) in the 6 months following emergency department visits. VA quickly implemented across the entire VA health care system and SPED was born. When a veteran presents to the emergency department or an urgent care center and is assessed as being at risk of suicide, but still safe enough to be discharged home, VA deploys a suicide safety planning intervention, including lethal means safety counseling, while the person is still in the emergency department or urgent care center. After discharge, the individual veteran is then personally contacted through regular outreach calls to facilitate engagement in outpatient mental health care. Ongoing calls are made until the Veteran is engaged in mental health care. Currently SPED is now being rolled out across all 140 health care systems.

### Recovery Engagement and Coordination for Health—Veterans Enhanced Treatment (REACH-VET)

In collaboration with the National Institute of Mental Health, VA developed REACH-VET, a clinical program based upon VA's electronic health record system using predictive analytics to identify veterans at the highest statistical risk for suicide in order to engage outreach and prevention efforts (9, 10). Monthly, local points of contact receive list of veterans deemed to be at highest risk for suicide. Clinical providers then provide personal outreach to each individual veteran to ensure all needed care is provided and treatment plans are reviewed (11). Initial validation studies highlight how this approach identifies veterans with 30–60 times higher rate for suicide, providing a potential mechanism for earlier intervention prior to a time of crisis. Full program evaluation efforts are underway to continue to study outcomes from this national rollout. Progress is tracked monthly and a technical assistance is provided to facilities facing challenges in implementation.

### Same Day Access

Connecting veterans to care the same day as services is needed is a critical component of suicide prevention. As part of the My VA Access Initiative, VA established same day mental health and primary care services across the nation in 2017. The My VA Access Initiative also included a larger emphasis on expanded implementation of Primary Care Mental Health Integration (PCMHI) which is one method of providing same day access to mental health services as part of routine primary care, reducing stigma, and increasing timeliness of service delivery. PCMHI has been shown to reduce wait times for mental health services, increase odds of attending future appointments, and lower no-show rates for appointments (12–15). It also provides an opportunity to deliver mental health services to those who may otherwise not seek them and to identify, prevent, and treat mental health conditions at the earliest opportunity. This is an important ingredient in suicide prevention because research has shown that 45% of individuals (16) who die by suicide have contact with a primary care provider (PCP) in the month prior to their death.

### Veterans Crisis Line

In addition to providing same day access to services at VA facilities, the Veterans and Military Crisis Line (VMCL) connects Veterans in crisis with qualified, caring VA responders through a confidential toll-free hotline, online chat, or text 24 h/7days/week. VMCL engages ~1,850 calls per day, sees an additional 300 contacts through chat and text programs, and offers ~360 referrals per day to local VA Suicide Prevention Coordinators who contact Veterans to ensure continuity of care with local VA providers. VMCL consistently exceeds performance targets. In 2019, VCL responders answered 96.82% of calls in 20 s or less with an average speed of 9.92 s, maintained an abandonment rate of 2.78%, had a rollover rate of 0.027% which was a 98% reduction in rollovers from FY18.

## Examples of Va's Community-Based Prevention Strategies

Of the 17 veterans who die by suicide every day, nine have never received VHA services and two have not received VHA services within the last 2 years. Moving upstream and reaching outside VA's walls to engage veterans in the community in lifelong health, well-being, and resilience is a critical part of VA's National Strategy. Community prevention focuses addressing social determinants of health outside the VHA healthcare system to promote early awareness and prevention prior to times of crisis, while also expanding collaboration and coordination of services across all veterans, families, Non-VHA healthcare systems, other community partners, and the VA. Community-based interventions are science-based approaches to changing community systems and contexts to improve population health outcomes (17), and these have been shown to effectively reduce suicide rates in diverse communities around the world (18). Three examples of community prevention models that have shown promise, Governor/ Mayoral Challenges VISN Community Prevention Pilots, and Together with Veterans are outlined below. VA is currently actively deploying all three and supports them with technical assistance. The interrelation among these programs is seen in the figure.

### Governor's and Mayor's Challenges

In 2018, VA partnered with the Substance Abuse and Mental Health Service Administration (SAMHSA) to launch Mayor's Challenge and in 2019 it expanded these efforts to launch the Governor's Challenges. These challenges engage both government and community partners in the development of regionally developed interagency strategic plans to address veteran suicide through the deployment of evidence-based strategies. The Mayor's Challenge currently consists of 24 cities and counties. Since the program's inception in 2019, seven states have joined the Governor's Challenges and the program is expanding to 28 additional states over the next 2 years and then with a final goal of engaging all 50 states. City and states are provided with technical assistance and support through site visits, policy academies, and virtual consultation to enhance their plans and incorporate evidence-based strategies to reach out to all veterans in their local areas to prevent suicide, pairing state-level policy makers with local leaders to implement comprehensive plans.

### VISN Community Prevention Pilots

Over the past year, the Office of Mental Health and Suicide Prevention partnered with Veterans Integrated Service Network (VISN) 23 in developing a pilot program to promote community prevention strategies to reach veterans through community engagement and partnerships focused upon coalition building at the local level. Implementation scientists from the University of Pittsburgh's Program Evaluation and Research Unit (PERU) and VA leadership worked collaboratively to provide technical assistance and facilitation hire and support 10 Community Engagement and Partnership Coordinators (CEPCs). CEPCs supplement the work of VA's 450+ suicide prevention coordinators by focusing on expanding community efforts to increase awareness of veteran suicide and moving awareness to engagement of local coalitions to implement community-focused evidence-based suicide prevention strategies. The CEPCs will collaborate at the community, regional, and state levels, to implement community partnerships, Together with Veterans, and the Governor's Challenge. Program evaluation efforts are now underway with planned expansion to three other VISNs this year, with an ultimate goal of engaging all 18 VISNs.

### Together With Veterans (TWV)

TWV is a community-based suicide prevention program for rural Veterans (19), which is focused upon partnering rural veterans and their communities to implement community-based suicide prevention. Based in implementation science, TWV assists veterans in the community in implementing evidence-based suicide prevention strategies to reach rural veterans. TWV is a VA Office of Rural Health program focused on empowering and supporting Veteran to Veteran coalition building. This includes efforts to increase lethal means safety, gatekeeper training, training of primary care providers, stigma reduction and help-seeking behavior promotion, increasing access to crisis services, and enhancing efforts to support veterans at highest risk for suicide (19). Currently, TWV is deployed in several rural regions with additional sites being added the 2020 calendar year.

## Discussion

Suicide prevention is the top clinical priority for VA and a priority for public health across the globe. VA has embarked on a comprehensive program of clinically-based and community-based strategies within a public health framework guided by the research currently available. The above strategies are just a few examples of VA's overarching plan to employ a public health model in the deployment of Suicide Prevention 2.0 over the coming decade combined with specific strategies for implementation now to not only prevent death, but engage Veterans on a journey of health, well-being, and resilience throughout the course of their lifetime. A critical aspect of Suicide Prevention 2.0 is a simultaneous, comprehensive evaluation of its impact, addressing quality, accountability, integrity, and effectiveness, and VA is committed to a transparent assessment and how this compares with initiatives in other sectors. Although suicide prevention is a core responsibility for VA and for all healthcare systems, the mission of suicide prevention cannot be fully achieved by any system. This is an urgent matter that requires a broad public response but one that is adapted to each individual circumstance. There will never be a “one and done” solution. The programs and initiatives outlined in this paper represent the current iteration of VA's committed effort to prevent suicide, but there is much more to learn and to do and for that we need to respond as a nation in recognition and gratitude for the service given by all those who are veterans.

## Author Contributions

DC: principal author, outlined, and drafted manuscript. LK and MM: edited and contributed content. All authors contributed to the article and approved the submitted version.

## Conflict of Interest

The authors declare that the research was conducted in the absence of any commercial or financial relationships that could be construed as a potential conflict of interest. The reviewer AEM declared a shared affiliation with the authors to the handling editor at time of review.
